# T-cell activation decreases miRNA-15a/16 levels to promote MEK1–ERK1/2–Elk1 signaling and proliferative capacity

**DOI:** 10.1016/j.jbc.2022.101634

**Published:** 2022-01-25

**Authors:** Frank Urena, Chi Ma, FuKun W. Hoffmann, Lance G.A. Nunes, Johann Urschitz, Stefan Moisyadi, Vedbar S. Khadka, Youping Deng, Peter R. Hoffmann

**Affiliations:** 1 Department of Cell and Molecular Biology, John A. Burns School of Medicine, University of Hawaii, Honolulu, Hawaii, USA; 2 Department of Molecular Biosciences and Bioengineering, University of Hawaii at Manoa, Honolulu, Hawaii, USA; 3 Department of Anatomy, Physiology and Biochemistry, John A. Burns School of Medicine, University of Hawaii, Honolulu, Hawaii, USA; 4 Department of Quantitative Health Sciences, John A. Burns School of Medicine, University of Hawaii, Honolulu, Hawaii, USA

**Keywords:** miRNA, mitogen-activated protein kinase, cell cycle, cell growth, cell signaling, CFSE, carboxyfluorescein succinimidyl ester, DOX, doxycycline, ERK1/2, extracellular signal–regulated protein kinase 1/2, FDR, false discovery rate, MEK1, mitogen-activated protein kinase kinase 1, MS, mass spectrometry, OT-I, mice with Tcrα-V2/Tcrβ-V5 transgenic inserts, TCR, T-cell receptor, TMT, tandem mass tag

## Abstract

While miRs have been extensively studied in the context of malignancy and tumor progression, their functions in regulating T-cell activation are less clear. In initial studies, we found reduced levels of miR-15a/16 at 3 to 18 h post–T-cell receptor (TCR) stimulation, suggesting a role for decreased levels of this miR pair in shaping T-cell activation. To further explore this, we developed an inducible miR15a/16 transgenic mouse model to determine how elevating miR-15a/16 levels during early stages of activation would affect T-cell proliferation and to identify TCR signaling pathways regulated by this miR pair. Doxycycline (DOX)-induced expression of miR-15a/16 from 0 to 18 h post-TCR stimulation decreased *ex vivo* T-cell proliferation as well as *in vivo* antigen-specific T-cell proliferation. We also combined bioinformatics and proteomics approaches to identify the mitogen-activated protein kinase kinase 1 (MEK1) (*Map2k1*) as a target of miR-15a/16. MEK1 targeting by miR-15a/16 was confirmed using miR mimics that decreased *Map2k1* mRNA containing the 3′-UTR target nucleotide sequence (UGCUGCUA) but did not decrease *Map2k1* containing a mutated control sequence (AAAAAAAA). Phosphorylation of downstream signaling molecules, extracellular signal–regulated protein kinase 1/2 (ERK1/2) and Elk1, was also decreased by DOX-induced miR-15a/16 expression. In addition to MEK1, ERK1 was subsequently found to be targeted by miR-15a/16, with DOX-induced miR-15a/16 reducing total ERK1 levels in T cells. These findings show that TCR stimulation reduces miR-15a/16 levels at early stages of T-cell activation to facilitate increased MEK1 and ERK1, which promotes the sustained MEK1–ERK1/2–Elk1 signaling required for optimal proliferation.

T-cell lymphocytes are activated through the T-cell receptor (TCR) together with coreceptors such as CD28, which triggers cellular proliferation as a key step in promoting adaptive immunity (1). Mitogen-activated protein kinase pathways are rapidly activated downstream of TCR engagement, and one of these pathways of particular importance for promoting proliferative capacity is the Ras/Raf/mitogen-activated protein kinase kinase (MEK)/extracellular signal–regulated protein kinase (ERK) pathway (2). TCR engagement leads to rapid activation of Ras, which in turn activates RAF kinase, which in turn phosphorylates and activates MEK1 and MEK2 (mitogen-activated protein kinase kinase 1 and 2). MEK1/2 phosphorylates and activates ERK1/2, which subsequently phosphorylates cytoplasmic and nuclear substrates involved in the regulation of cell proliferation. Even though this pathway is activated within minutes of TCR engagement to initiate T-cell activation, some degree of sustained MEK–ERK activity in later stages is required for cells to upregulate gene transcription that facilitates cell cycle entry and suppresses negative regulators of the cell cycle (3). In fact, interleukin 2 that is secreted after initial MEK–ERK pathway activation has been shown to stimulate MEK1 expression in later stages of activation (4). This suggests that MEK1 levels increasing well after initial TCR signaling may be an important contributor to the sustained activation of T cells that promote cell cycling and proliferation.

An important role in regulating T-cell activation is emerging for miRs, which are short noncoding RNAs that mostly bind through base pairing to 3′-UTRs of target mRNAs to decrease target protein levels (5, 6, 7). The magnitude of target protein reduction is usually modest, but the accumulated effects on signaling pathways are often important for shaping cellular functions (8). T-cell activation has been shown to induce dynamic changes in the expression of miRs, some of which increase, whereas others decrease to shape T-cell activation and differentiation (9). The miR-15/16 family of miRNAs has been extensively studied for their roles as tumor suppressors (10), but evidence is emerging for this miR pair as regulators of T-cell activation (11). The miR-15/16 family consists of two loci present in the human and mouse genome, one of which is located on chromosome 13 in humans and 14 in mice, which is comprised of a contiguous DNA sequence encoding immature mir-15a and mir-16-1 separated by a 54-nucleotide spacer. RNA transcribed from this DNA sequence generates two immature hairpin RNAs (mir-15a; mir-16-1) that are processed into four mature miRs (miR-15a-3p and miR-15a-5p; miR-16-1-3p and miR-16-1-5p). The two mature -5p miRs (referred to as miR-15a/16 hereafter) share sequence homology and simultaneously target several mRNAs (12).

A recent study using a loss-of-function approach showed that miR-15a/16 directly targeted mRNA that were part of an extensive network of pathways that influence T-cell differentiation, survival, and memory (11). Interestingly, decreased miR15a/16 levels were detected 1 to 4 days post-TCR stimulation, but it was not determined how impaired downregulation of this miR cluster may affect T-cell activation occurring during the critical early stages as these cells prepare to proliferate. In other words, if miR-15a/16 levels cannot sufficiently decrease during early T-cell activation, how would proliferation be impacted and which signaling pathways would be most affected? To address these questions, we developed a gain-of-function model: an inducible miR15a/16 transgenic mouse in which levels of these miRs in T cells are elevated compared with endogenous levels during early stages of TCR-induced activation. Higher miR-15a/16 levels during TCR activation were found to impair T-cell proliferation *ex vivo* and *in vivo*. Proteomics, bioinformatics, and Western blot analyses identified MEK1 and ERK1 as direct targets of miR15/a16, and further studies showed that the MEK1–ERK1/2–Elk1 pathway was regulated by miR15a/16 during T-cell activation. These findings reveal a new mechanism by which decreased miR-15a/16 during TCR activation promotes sustained signals important for T-cell proliferation.

## Results

### Inducing higher miR-15/16 levels during T-cell activation leads to decreased proliferation

**Figure 1:**
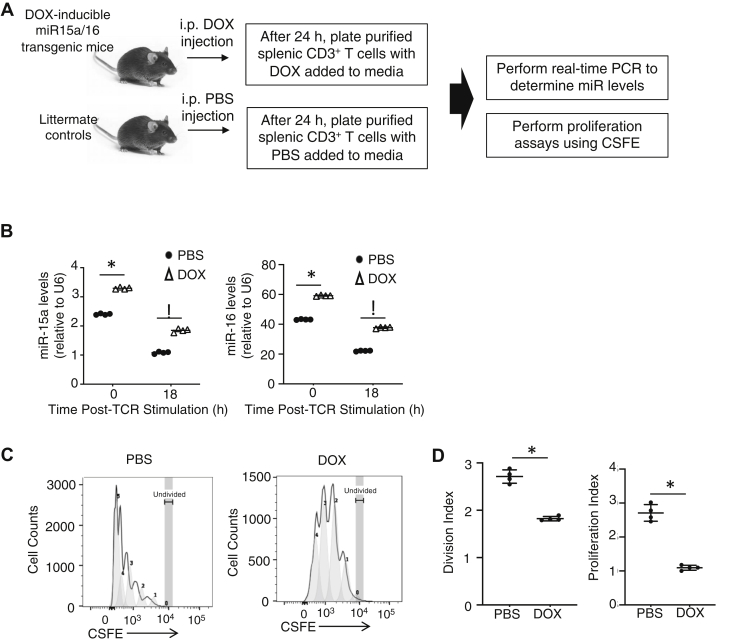
**Inducing higher levels of miR-15a/16 during T-cell activation decreases proliferation.***A*, experimental design for mice containing a DOX-inducible miR-15a/16 transgene. *B*, CD3^+^ T cell purified from transgenic mice treated either with DOX or PBS (control) was not stimulated or TCR stimulated for 18 h followed by real-time PCR analyses of miR-15a and miR-16 levels relative to U6 miR (housekeeping). *C* and *D*, CFSE-loaded transgenic T cells were treated with DOX or PBS and activated for 72 h followed by flow cytometric analysis. Because levels of CFSE fluorescence decreases as cells divide, this allowed calculation of division index and proliferation index as described in the Experimental procedures section. Means of replicates (N = 4/mouse) were compared using a Student’s *t* test and expressed as mean ± SD with ∗*p* < 0.05. CFSE, carboxyfluorescein succinimidyl ester; DOX, doxycycline.

Because a recent report showed that miR-15a/16 levels decreased over several days after T-cell activation (11), we set out to determine how interfering with this decrease would affect T-cell proliferation. To induce miR-15a/16 levels higher than endogenous levels, we generated a transgenic mouse model with miR-15a/16 expression under the control of doxycycline (DOX) treatment. These transgenic mice were injected once with DOX or PBS as a control, and after 24 h, splenic CD3^+^ T cells were purified and either frozen at −20 ^°^C (nonactivated) or TCR activated for 18 h in media containing DOX or PBS followed by freezing at −20 ^°^C (Fig. 1*A*). Real-time PCR was used to measure relative levels of miR-15a and miR-16, and results showed that DOX induced ∼35% and ∼70% higher levels of both miRs in nonactivated and activated T cells, respectively (Fig. 1*B*). Carboxyfluorescein succinimidyl ester (CFSE) assays were conducted on *ex vivo* T cells to analyze the proliferative capacity in conditions of elevated miR-15a/16 compared with controls. Results showed that the division index (average number of cell divisions, including the undivided peak) and the proliferation index (total number of divisions divided by the number of cells that went into division) were both decreased in the DOX-treated group (Fig. 1*C*). As expected, DOX treatment did not affect the proliferative capacity of WT T cells (Fig. S1), suggesting that the elevated miR-15a/16 expression induced by DOX in the transgenic T cells, and not DOX itself, led to reduced proliferation.

**Figure 2:**
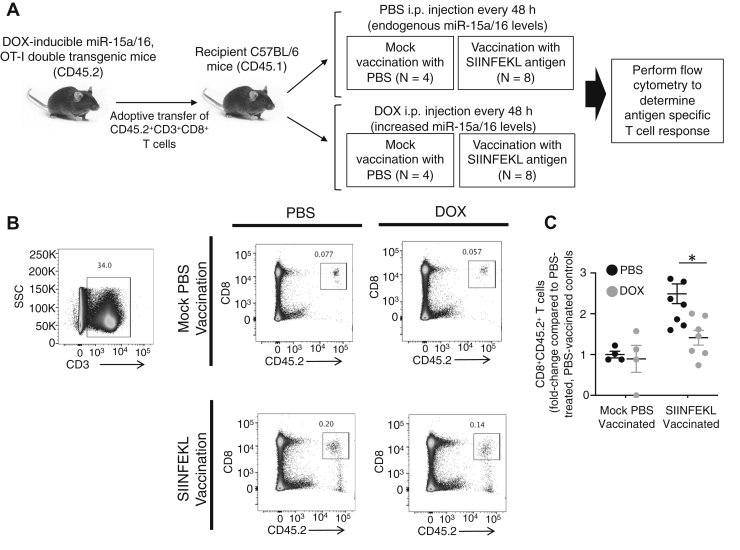
**Inducing higher levels of miR-15a/16 decreases *in vivo* antigen-specific proliferation of T cells.***A*, experimental design for adoptive transfer and antigen challenge with OVA peptide (SIINFEKL), followed by measuring T-cell responses for OVA-specific CD8^+^ T cells. *B*, flow cytometric analysis included identification of splenic CD3^+^ cells, followed by CD8^+^CD45.2^+^ cells. *C*, each group is expressed as fold change compared with the PBS-treated/PBS-vaccinated controls. Means of replicates (N = 4 mock vaccination; N = 7 SIINFEKL vaccination) were compared using a Student’s *t* test and expressed as mean ± SD with ∗*p* < 0.05. OVA, ovalbumin.

To evaluate the effects of elevated miR-15a/16 on *in vivo* T-cell proliferation, a double transgenic mouse model was generated by crossing the DOX-inducible miR-15a/16 mice with Tcrα-V2/Tcrβ-V5 transgenic mice (OT-I). The CD8^+^ T cells from these double transgenic mice express a TCR recognizing the ovalbumin peptide residues 257 to 264 (SIINFEKL) in the context of H2-K^b^, along with the DOX-inducible miR-15a/16 transgene. Adoptive transfer of purified CD8^+^ T cells from these double transgenic mice that contained the CD45.2 isoform of the common leukocyte antigen into CD45.1 recipient mice followed by immunization with the OVA257 to 264 peptide allowed measurement of *in vivo* expansion of the CD45.2^+^ antigen-specific T cells without or with DOX-induced miR-15/16 expression (Fig. 2*A*). Results showed that DOX treatment led to significantly lower expansion of the antigen-specific CD8^+^ T cells in response to SIINFEKL vaccination (Fig. 2, *B* and *C*). Consistent with the *ex vivo* data described previously, these *in vivo* findings suggest that elevated miR-15/16 levels in CD8^+^ T cells interfere with proliferation in response to antigen challenge.

### Identification of MEK1 as a target of miR-15a/16 during T-cell activation

We sought to identify signaling molecules and pathways that depend on decreased miR-15a/16 levels in T cells during TCR-induced activation. Using the DIANA-miRPath prediction program, we identified 72 mRNAs potentially targeted by miR-15a and miR-16 with a cutoff score of 0.8 (1.0 being the strongest) that were categorized in the Kyoto Encyclopedia of Genes and Genomes T-cell signaling pathway (Fig. S2). In combination with this analysis, we performed discovery phase proteomics to measure levels of >6000 proteins in activated T cells from the transgenic mice treated with DOX to induce expression of miR-15a/16 compared with PBS controls (Table S1). Combining the data from these two approaches identified those proteins with levels changed by DOX treatment that also were predicted to be directly targeted by miR-15a/16 (Table 1). Five proteins were identified, but only MEK1 was decreased by DOX treatment with an adjustable *p* value that indicated statistical significance. Interestingly, the DIANA-miRPath database suggested that MEK1 was not an experimentally supported target of miR-15a/16, so we next investigated the direct targeting of MEK1 by miR-15a/16.

**Table 1:** TCR signaling proteins identified as miR-15a/16 targets *via* bioinformatics and proteomics

Symbol	Protein name	Prediction score	Experimentally supported?	Activated Tg T cells, DOX/no DOX	*p*	Adjusted *p*
miR-15a						
PiK3r1	Phosphatidylinositol 3-kinase regulatory subunit alpha	0.971	Yes	−12.3%	0.13	0.34
Map2k1	MEK1	0.959	No	−24.7%	0.0005∗	0.002∗
NfatC3	Nuclear factor of activated T-cells, cytoplasmic 3	0.959	No	−3.0%	0.59	0.77
CD28	CD28 coreceptor	0.921	Yes	37.0%	0.005	0.071
Sos2	Son of sevenless homolog 2	0.851	Yes	18.4%	0.076	0.25
miR-16						
PiK3r1	Phosphatidylinositol 3-kinase regulatory subunit alpha	0.969	Yes	−12.3%	0.13	0.34
Map2k1	MEK1	0.948	No	−24.7%	0.0005∗	0.026∗
NfatC3	Nuclear factor of activated T-cells, cytoplasmic 3	0.965	No	−3.0%	0.59	0.77
CD28	CD28 coreceptor	0.893	Yes	37.0%	0.005	0.071
Sos2	Son of sevenless homolog2	0.895	Yes	18.4%	0.076	0.25

∗ Statistically significant.

**Figure 3:**
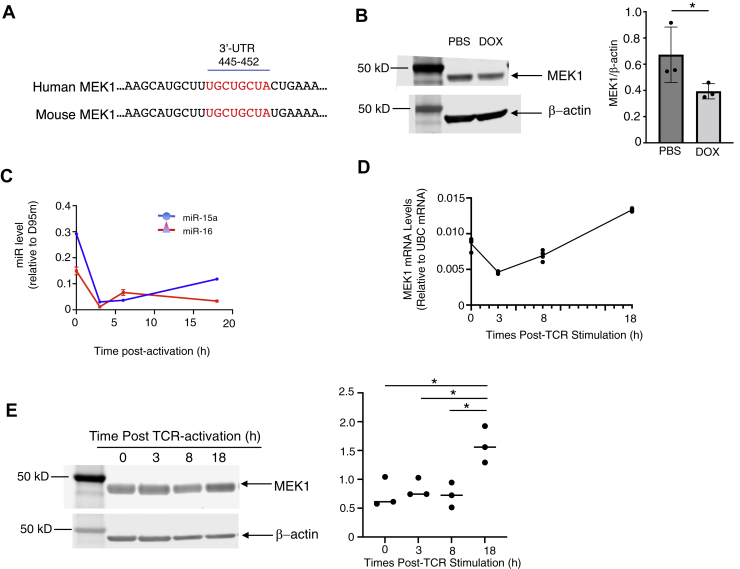
**Changes in miR-15a/16 levels oppose MEK1 levels in T cells.***A*, human and mouse mRNA sequences (NM_002755.4; NM_008927.4) contain a consensus 3′-UTR miR-15a/16 binding site (*red*). *B*, Western blot of TCR activated for 18 h shows lower MEK1 levels in transgenic cells treated with DOX to induce miR-15a/16 compared with PBS-treated controls. Loading control used was β-actin. Densitometry was performed on biological repeats shown in Fig. S3. *C*, real-time PCR shows relative levels of miR-15a and miR-16 decreased over time in WT T cells stimulated through the TCR. *D*, real-time PCR analyses of WT T cells activated over time show that MEK1 mRNA do not increase in early stages of TCR stimulation but are increased at 18 h post-TCR stimulation. *E*, Western blot shows levels of MEK1 increased at 18 h in WT T cells stimulated through the TCR. Densitometry was performed on biological repeats shown in Fig. S3. Data are shown as mean ± SD (N = 4 for *C* and *D*; N = 3 for *B* and *E*), which were compared using a Student’s *t* test and one-way ANOVA followed by Tukey’s post-test, respectively, with ∗*p* < 0.05. DOX, doxycycline; MEK1, mitogen-activated protein kinase kinase 1; TCR, T-cell receptor.

Examination of the 3′-UTRs of human and mouse MEK1 mRNA revealed a conserved putative miR-15a/16 binding site for both species (Fig. 3*A*). Western blot analysis of activated T cells purified from the miR-15a/16 transgenic mice showed that DOX-induced miR-15a/16 expression led to decreased MEK1 (Fig. 3*B*). Consistent with these data, the decreased levels of miR-15a/16 observed upon TCR-induced T-cell activation both preceded and opposed increased MEK1 mRNA and protein levels (Fig. 3, *C*–*E*).

**Figure 4:**
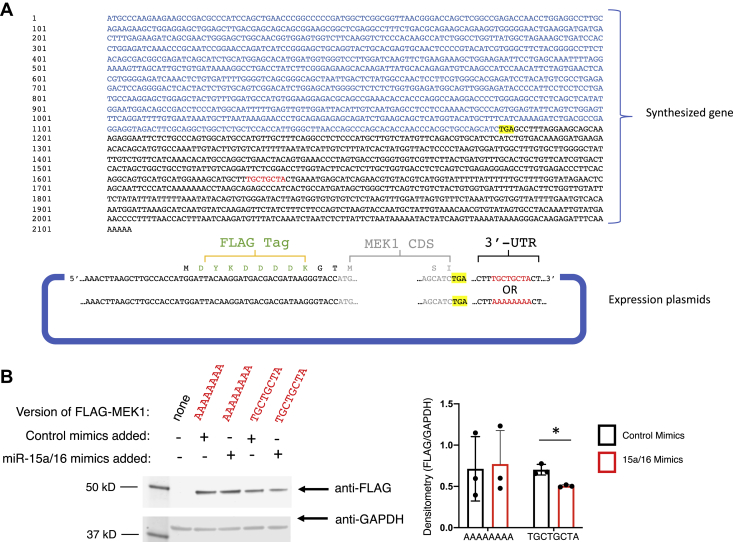
**The 3′-UTR target sequence in MEK1 is recognized by miR-15a/16.***A*, a gene encoding MEK1 was synthesized containing the predicted target nucleotide sequence (TGCTGCTA) or mutated sequence (AAAAAAAA) and ligated into a FLAG-tagged expression plasmid. *B*, Western blot was used to analyze expression in HEK293 cells of FLAG-MEK1 from each plasmid cotreated with miR-15a/16 mimics or nontargeting control mimics, with GAPDH as a loading control and biological replicates used for densitometry shown in Fig. S3. Means of replicates (N = 3) were compared using a Student’s *t* test and expressed as mean ± SD with ∗*p* < 0.05. HEK293, human embryonic kidney 293 cell line; MEK1, mitogen-activated protein kinase kinase 1.

To determine if the predicted mRNA motif (3′-UTR 445–452) serves as the target for the miR-15a/16, we synthesized two different versions of MEK1 encoding DNA sequences: one containing the WT 3′-UTR UGCUGCUA target sequence and another containing this sequence mutated to 3′-UTR AAAAAAAA, which were then each ligated into N-terminal FLAG-tagged expression plasmids (Fig. 4*A*). Human embryonic kidney 293 cells were transfected with these plasmids along with functional mimics for miR-15a/16 or control nontargeting mimics. Levels of MEK1 were analyzed by Western blot, and results showed that addition of miR-15a/16 mimics led to decreased MEK1 levels for cells transfected with WT MEK1 construct but had no effect on MEK1 levels for the mutated MEK1 construct (Fig. 4*B*). While the reduction was modest (−28%), this is consistent with typical reduction by miRs and with data from Table 1 and Figure 3. Successful uptake of the fluorescein-tagged miR mimics was confirmed by fluorescent microscopy, and biological replicates included in densitometry are included in Fig. S4. Overall, these data suggest that the predicted 3′UTR motif of MEK1 mRNA (UGCUGCUA) functions as a miR-15a/16 target for suppressing its expression, consistent with opposing levels of each observed during T-cell activation.

### The effects of DOX induced miR-15/16 expression on the MEK1–ERK1/2–Elk1 pathway

**Figure 5:**
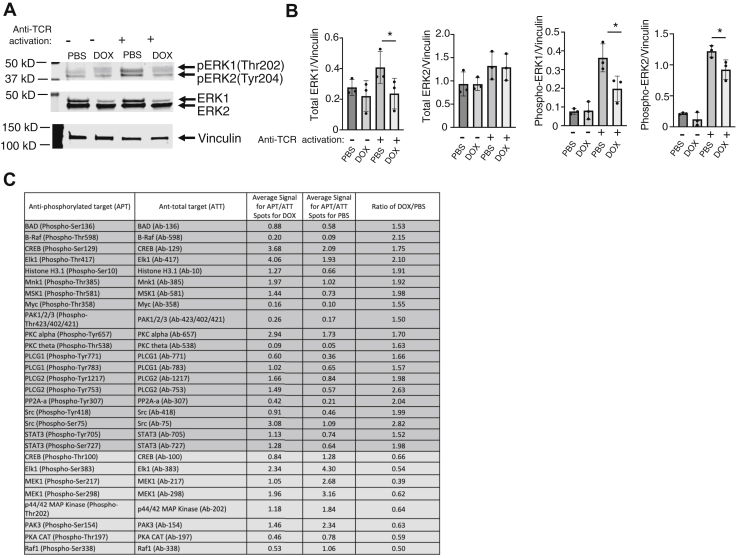
**ERK1/2 phosphorylation is decreased with DOX-induced miR-15a/16.***A*, Western blot analysis of p-ERK1(Thr202) and p-ERK2(Tyr204) along with total ERK1 and ERK2 in unstimulated and 18 h TCR-stimulated T cells treated with PBS or DOX, with β-actin serving as a loading control. *B*, densitometry was performed on biological repeats shown in Fig. S6. *C*, lysates from 18 h TCR-stimulated T cells were used to conduct an ERK signaling phospho antibody array to compare DOX- *versus* PBS-treated cells for levels of phosphorylated amino acid residues on different signaling proteins. *Darker rows* indicate increased signals with DOX treatment, and *lighter rows* indicate decreased signals. Mean of each p-residue signal (N = 6) was normalized to mean of total residue signal (N = 6). DOX, doxycycline; ERK1/2, extracellular signal–regulated protein kinase 1/2; TCR, T-cell receptor.

Since MEK1 is a dual specificity kinase that phosphorylates and activates ERK1 and ERK2 by phosphorylation on threonine (Thr) and tyrosine (Tyr) residues (13), we analyzed the effect of DOX-induced miR-15a/16 expression on ERK1/2 phosphorylation during T-cell activation. Western blot analysis using an antibody recognizing both p-ERK1(Thr202) and p-ERK2(Tyr204) showed that DOX-induced expression of miR-15a/16 reduced phosphorylation of both residues in activated T cells (Fig. 5, *A* and *B*). Importantly, control experiments using WT T cells showed that DOX treatment had no effect (Fig. S5). Surprisingly, total ERK1 also decreased with DOX treatment for unstimulated and TCR-stimulated T cells. Our bioinformatics analyses shown in Fig. S2 listed ERK1 (mitogen-activated protein kinase 3) as a lower scored, predicted target for miR-15a (score = 0.668) and miR-16 (0.658). The proteomics data in Table S1 showed that DOX-induced miR-15a/16 decreased ERK1 by 22% with a significant *p* value for the individual moderated *t* test (*p* = 0.0048) but nonsignificant at the false discovery rate (FDR)-adjusted *p* value corrected for multiple testing (*p* = 0.067). Importantly, DOX-induced expression of miR15a/16 had no effect on p-ERK1/2 in the early stages (0, 30, and 60 min) of TCR stimulation (Fig. S6), which is consistent with the late stage increase in p-ERK1/2 discussed previously resulting from increased MEK1 protein levels. These results combined with the Western blot data suggest that the reduced miR-15a/16 observed with TCR stimulation is crucial for allowing sufficient levels of MEK1 and ERK1 along with p-ERK1/p-ERK2, revealing a double hit on the MEK1–ERK1 pathway by this miR pair.

**Figure 6:**
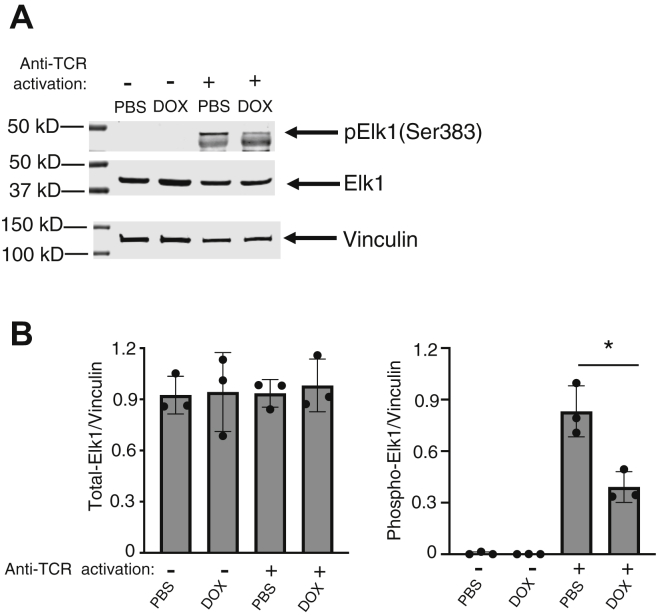
**Elk1 phosphorylation is decreased with DOX-induced miR-15a/16.***A*, Western blot analysis of p-Elk1(Ser383) and total Elk1 in unstimulated and 18 h TCR-stimulated T cells treated with PBS or DOX, with β-actin serving as a loading control. *B*, densitometry was performed on biological repeats of Western blots shown in Fig. S6. Means of replicates (N = 3) were compared using a Student’s *t* test and expressed as mean ± SD with ∗*p* < 0.05. DOX, doxycycline; TCR, T-cell receptor.

We next carried out an ERK signaling phospho antibody array to simultaneously analyze >100 phosphorylation sites on signaling molecules for identifying potential signaling molecules downstream from ERK1/2 that may be affected by DOX-induced miR-15a/16 expression. We identified 20 and 8 phosphorylation sites that were upregulated or downregulated, respectively, by ≥1.5-fold with DOX treatment in activated miR-15a/16 transgenic T cells (Fig. 5*C*). The downregulated p-Elk1(Ser383) in this list was of particular interest, given that phosphorylation of this residue on Elk1 by activated ERK1/2 promotes proliferation of cancer cells (14). Western blot analysis confirmed that DOX-induced miR-15a/16 decreased p-Elk1(Ser383) during T-cell activation (Fig. 6). Control experiments showed that DOX treatment had no effect on WT T cells with no miR-15a/16 transgene (Fig. S5). Total Elk1 was not affected by DOX, and no predicted miR-15a/16 target sequence was found in the mRNA sequence, suggesting that the effects on p-Elk1(Ser383) likely are due to reduced ERK1/2 activation caused by elevated miR-15a/16 levels.

## Discussion

The role of miR15a/16 in regulating tumor progression or malignancy for a variety of cancers has been well established (15, 16). Mechanistic data suggest that miR-15/16 can function as a tumor suppressor by directly targeting B-cell lymphoma 2 and affecting survival (17), but signaling molecules and pathways regulated by this miR pair in activated T cells may likely be different. With the recent report showing that miR-15a/16 levels decreased over several days after T-cell activation (11), this study set out to determine how this clustered set of miRs may target key signaling molecules in TCR-activated T cells. By generating a new transgenic mouse model in which we could induce higher levels of miR-15a/16 with DOX treatment, we were able to show that elevated levels of these miRs decreased the proliferative capacity of T cells. Moreover, by applying -omics and array approaches to evaluate signaling pathways regulated during T-cell activation, we established that MEK1 and ERK1 are directly targeted by miR15a/16 and the MEK1–ERK1/2–Elk1 pathway as an important proproliferation pathway that is regulated by this miR cluster.

Our data in WT T cells showing that miR-15a/16 decreased after TCR activation confirm the previous results of Gagnon *et al.* (11) and extends their data by showing a very early decrease in these miRs at ∼3 h post-TCR activation. The increased MEK1 is not evident until 18 h post-TCR stimulation, suggesting that it takes several hours for the effects of lower miR levels to impact MEK1 protein levels. The 18-h post-TCR stimulation time point is well after the period of ∼30 min that TCR-induced Ras–Raf–MEK–ERK pathway is conventionally considered for promoting optimal T-cell activation. For example, MEK1 inhibitors such as U0126 are most often added 1 h prior to TCR stimulation in order to interrogate roles for this kinase in activation (18, 19). Therefore, the role of TCR-induced miR-15a/16 reduction in allowing MEK1 protein to increase 18 h post-TCR activation is affecting a sustained MEK–ERK signal that is important for optimal T-cell proliferation.

Elk1 has been demonstrated to be a key signaling molecule downstream of ERK1/2 playing a role in the regulation of a wide range of normal cellular functions (20, 21). Transcriptional activation regulated by p-Elk1(Ser383), in particular, plays a role in a variety of cellular stimuli, such as stress, inflammatory cytokines, and growth factors that shape the cellular responses and affect apoptosis, growth, proliferation, and differentiation (22, 23). Moreover, ERK1/2 activation is clearly important for phosphorylation of Elk1 at this residue. For example, the complexing of Elk1 with serum response factor following nerve growth factor stimulation was shown to be dependent on phosphorylation at Ser383 and Ser389 by ERK1/2 in PC-12 cells (24). Our data link miR-15a/16 with both ERK1/2 activation and p-Elk1(Ser383) during T-cell activation, which broadens the repertoire of cellular responses regulated by this versatile pathway.

Overall, the findings from our current study provide important insight into the role of miR-15a/16 on T-cell proliferation and pathways impacted by the dynamics of this miR pair post-TCR activation. As stated previously, in addition to the miR-15a/16-1 cluster (human chromosome 13 and mouse chromosome 14), a related miR-15b/16-2 cluster (human chromosome and mouse chromosome 3) targets similar mRNAs. This did not impact our gain-of-function experiments in which we induced expression of transgenic miR-15a/16 on top of any endogenous miRs present in the cells, but both miR clusters must be considered with loss-of-function studies or therapies designed to inhibit the targeting by one or the other of these miR clusters. For example, double knockout of the two miR-15/16 loci in mice resulted in the development of acute myeloid leukemia (25), which was more severe than in single locus miR-15a-16-1 knockouts (26). When designing therapeutics to treat different types of cancers by focusing on miR-15a/16, the effects on T-cell immunity should also be considered. Our data suggest that T-cell proliferation is only partially diminished by slightly increasing miR-15a/16 (∼20–30%), so effects on T-cell activation may not be as potent as effects on rapidly dividing cancer cells. However, side-by-side testing of a specific therapeutics on malignancy *versus* T-cell function would be informative.

## Experimental procedures

### Mice

The Institute for Biogenesis Research Transgenic Mouse Core at the University of Hawaii at Manoa used transposon-enhanced pronuclear injection to generate a stable DOX-inducible miR-15a/16 mouse line. The Origene plasmid clone MI0000564 SC400794 containing a transgene including the mature miR-15a sequence (uagcagcacauaaugguuugug) and miR-16 sequence (uagcagcacguaaauauuggcg) with a 54 bp spacer sequence between the two, similar to the miR-15a/16-1 locus found on mouse chromosome 14, was used as a template to amplify the desired miR-15a/16 region. The PCR product was then subcloned into a pENTR1A vector (Thermo Fisher Scientific) that included a luciferase reporter gene under a TRE-3G promoter, with the reverse tetracycline-controlled transactivator 3 driven by a human cytomegalovirus promoter. The miR-15a/16 sequence was situated in the 3′UTR of luciferase gene. These steps were all performed using restriction digestion and ligation cloning. In the final step, we recombined this miR-15a/16 pENTR1A vector with our pmhyGENIE-3 *piggyBac* vector. Transposase-enhanced pronuclear injection was performed as previously described (27, 28) using B57BL/6 and CD1 mice, purchased from Jackson Laboratories. Genomic DNA was isolated from pups, and genotyping PCR was performed. To determine the number of transgenes for the founder generation as well as F1 (crossed with WT B57BL/6), we performed transgene copy number assays by duplex Taqman real-time PCR as previously described (29), and results were analyzed using Applied Biosystems CopyCaller software. These mice were crossed with OT-I mice purchased from Jackson Laboratories (catalog no.: 003831) to generate double transgenic mice with two copies of the DOX-inducible miR-15a/16 transgene and one copy of the OT-I TCR transgene. Experiments using mice included age/sex-matched male and females 8 to 12 weeks of age with two copies of the transgene. DC45.1^+^ mice (catalog no.: 002014) were purchased from Jackson Laboratories. Animal protocols were approved by the University of Hawaii Institutional Animal Care and Use Committee.

### Antibodies and reagents

Antibodies for Western blots included anti-MEK1, anti-ERK1/2, and anti-p-ERK1/2 (catalog no.: 12671S, 1:1000 dilution; catalog no.: 9107S, 1:1000 dilution; catalog no.: 4370T, 1:500 dilution; Cell Signaling); anti-FLAG (catalog no.: F1804; 1:1000 dilution; Millipore Sigma), anti-GAPDH (catalog no.: ab9485; 1:1000 dilution; Abcam), anti-p-Elk1 (catalog no.: 43004; 1:1000 dilution; QED Bioscience), anti-Elk1 and anti-β-actin (catalog no.: SC-365876, 1:1000 dilution and SC-517582, 1:2000 dilution, respectively; Santa Cruz). BioLegend antibodies for flow cytometry used at manufacturer's recommended concentrations included FITC-anti-CD3 (catalog no.: 100306), APC-anti-CD8 (catalog no.: 100712), and PE-anti-CD45.2 (catalog no.: 109808). Dead cells were detected with BV421 viable dye (Invitrogen). Mimic Dharmacon miRIDIAN reagents for miR-15a and miR-16 (catalog nos.: CTM-535609 and CTM-535610) as well as negative control mimic (catalog no.: CTM-535611) were purchased with 5′-fluorescein and 3′-cholesterol modifications. DOX was purchased from Sigma.

### Cell purification and stimulation

The miR-15a/16 transgenic mice were injected i.p. with DOX at 5 μg/g 1 day prior to spleen/lymph node harvest. Control mice received PBS injections. For T-cell isolation, spleens and inguinal lymph nodes excised from mice were homogenized into a single cell suspension, which was applied to T-Cell Enrichment Columns (R&D Systems). Purity was >90% CD3^+^ viable cells as determined by flow cytometry using a BD LSRFortessa cell analyzer, and data were analyzed using FlowJo software (BD Life Sciences). T cells (10^6^) were seeded in 96-well plates precoated with BioLegend anti-CD3 (10 μg/ml) plus anti-CD28 (1 μg/ml). Cells were incubated for different periods in RPMI1640 media containing 10% Seradigm 1500-500 FBS (VWR). *Ex vivo* T cells from mice receiving the DOX injection were cultured in complete media containing 2 μg/ml DOX for continued expression of miR-15/16.

### Western blots, phosphorylation profiling, flow cytometry, and real-time PCR

Western blots were carried out as previously described using a Li-Cor Odyssey infrared imaging system (30). Densitometry of Western blot bands was performed using ImageJ (National Institutes of Health). The ERK Signaling Phospho Antibody Array (Full Moon Biosystems) was used to profile levels of phosphorylated ERK target proteins per manufacturer's instructions. Intracellular staining of p-ERK was performed per manufacturer's protocol using Alexa Fluor 647 anti-p-ERK1/2 (BioLegend) and analyzed using a BD Fortessa flow cytometer and FlowJo, version 10 software. To quantify miRs, total RNA was isolated using an Omega Bio-Tek Total RNA Kit II, and the miScript PCR Starter Kit (Qiagen) was used along with forward primers for miR-15a-5p (CAG TAG CAG CAC ATA ATG GT) or miR-16-5p (TAG CAG CAC GTA AAT ATT GGC G). The housekeeping miR for relative fluorescence was U6 or D95 provided in the miScript PCR Starter Kit. Real-time PCR was performed on a Roche LightCycler 480 II with the following conditions: 15 min at 95 ^°^C; 40 cycles consisting of 15 s at 95 ^°^C, 30 s at 55 ^°^C, 30 s at 70 ^°^C with fluorescence collected during extension. Real-time PCR for mRNA levels was carried out as previously described (30), and primers included mMEK1 forward: CGT ACA TCG TGG GCT TCT AC, and mMEK1 reverse: CAG AAC TTG ATC CAA GGA CCC.

### Adoptive transfer and vaccinations

CD8^+^ T cells were purified from miR-15a/16 OT-I double transgenic mouse spleens using the Miltenyi CD8^+^ T-Cell Isolation Kit and then were retro-orbitally injected (10^6^ cells per mouse) into CD45.1^+^ recipient mice. DOX was i.p injected into one of the double transgenic mice at 5 μg/g 1 day prior to cell isolation, and the same DOX injection was given to mice receiving those cells every other day until the end of the experiment. The control double transgenic mouse received PBS i.p. injection, and PBS injections were given to mice receiving those cells every other day. One day following adoptive transfer, the mice were vaccinated as follows: s.c. injections with 50 μl of complete Freund's adjuvant (Sigma) emulsified with SIINFEKL peptide (20 mg in 50 ml of PBS) given on day 0 and boosted on day 10 with emulsified incomplete Freund’s adjuvant plus peptide at the same concentration. On day 17, the mice were sacrificed, and their spleen cells were harvested, red blood cells were lysed using RBC lysis reagent (MBL International) as recommended, and the cells were washed with 1 ml of PBS. Following centrifugation at 150*g* for 5 min, the media were aspirated, and the cells were resuspended in flow cytometry staining buffer and stained with fluorochrome-conjugated antibodies as recommended by manufacturers. Flow cytometry was performed using a BD LSRFortessa cell analyzer, and the data were analyzed using FlowJo software.

### Proteomics and bioinformatics

Frozen cell pellets (15 × 10^6^) were submitted to the UAMS Bioinformatics Core for tandem mass tag (TMT; Thermo Fisher Scientific) labeling of tryptic peptides for quantitative multiplexing. Proteins were reduced, alkylated, and purified by chloroform–methanol extraction prior to digestion with sequencing grade modified porcine trypsin (Promega). Tryptic peptides were labeled using TAG isobaric labeling reagents (Thermo Fisher Scientific) following the manufacturer’s instructions and combined into one 6-plex sample group. These reagents were prepared: buffer A = 0.1% formic acid, 0.5% acetonitrile; buffer B = 0.1% formic acid, 99.9% acetonitrile; both buffers adjusted to pH 10 with ammonium hydroxide for offline separation. The labeled peptide multiplex was separated into 46 fractions on a 100 × 1.0 mm Acquity BEH C18 column (Waters) using an UltiMate 3000 UHPLC system (Thermo Fisher Scientific) with a 50 min gradient from 98:2 to 60:40 buffer A:B ratio under basic pH conditions and then consolidated into 18 superfractions. Each superfraction was then further separated by reverse-phase XSelect CSH C18 2.5 um resin (Waters) on an in-line 150 × 0.075 mm column using an UltiMate 3000 RSLCnano system (Thermo Fisher Scientific). Peptides were eluted using a 60 min gradient from 98:2 to 60:40 buffer A:B ratio. Eluted peptides were ionized by electrospray (2.2 kV) followed by mass spectrometric analysis on an Orbitrap Eclipse Tribrid mass spectrometer (Thermo Fisher Scientific) using multinotch MS3 parameters with real-time search enabled. MS data were acquired using the Fourier transform mass spectrometry (MS) analyzer in top-speed profile mode at a resolution of 120,000 over a range of 375 to 1500 *m/z*. Following collision-induced dissociation activation with normalized collision energy of 35.0, MS–MS data were acquired using the ion trap analyzer in centroid mode and normal mass range. Using synchronous precursor selection, up to 10 MS/MS precursors were selected for higher collision dissociation activation with normalized collision energy of 65.0, followed by acquisition of MS3 reporter ion data using the Fourier transform MS analyzer in profile mode at a resolution of 50,000 over a range of 100 to 500 *m/z*. Proteins were identified, and MS3 reporter ions were quantified using MaxQuant (version 1.6.17.0; Max Planck Institute) against the UniProtKB *Mus musculus* (April 2020) database with a parent ion tolerance of 3 ppm, a fragment ion tolerance of 0.5 Da, and a reporter ion tolerance of 0.003 Da. Scaffold Q + S (Proteome Software) was used to verify MS/MS-based peptide and protein identifications (protein identifications were accepted if they could be established with less than 1.0% false discovery and contained at least two identified peptides; protein probabilities were assigned by the Protein Prophet algorithm [*Anal. Chem.* 75: 4646–58 (2003)]) and to perform reporter ion–based statistical analysis. Protein TMT MS3 reporter ion intensity values are assessed for quality using ProteiNorm, a tool for a systematic evaluation of normalization methods (31). The intensities were normalized using cyclic Loess normalization since it had the highest intragroup correlation and the lowest variance. The normalized data were used to perform statistical analysis using linear models for microarray data (limma) with empirical Bayes smoothing to the standard errors (32). Proteins with an FDR-adjusted *p* value <0.05 and a fold change of >2 were considered significant. Three PBS control cell pellets were compared with three DOX-induced T-cell pellets after 18 h of TCR stimulation, with data reported as the log_2_ fold change showing quantitative difference between PBS and DOX groups. For bioinformatics, DIANA-miRPath, version 3 web server (www.microrna.gr) (33) was used to predict the miRNA targets for four miRNAs: mmu-miR-16-5p, mmu-miR-15a-5p, mmu-miR-16-1-3p, and mmu-miR-15a-3p, based on DIANA-microT-coding DNA sequence algorithm. The algorithm computed a miRNA–gene interaction score for predicted targets in coding DNA sequence or 3′-UTRs. The genes with significant interaction score of above 0.8 were selected and further used to identify significantly enriched Kyoto Encyclopedia of Genes and Genomes pathways.

### Cell proliferation assays and *in vivo* antigen challenge

Mouse primary CD3^+^ T cells were negatively purified using Mouse Pan T-Cell Isolation Kit (Miltenyi), and 10^6^ cells were resuspended in 1 ml of cold PBS. Cells were loaded with 1 μl of 5 mM CellTrace CFSE (Thermo Fisher Scientific) in dimethyl sulfoxide and incubated at room temperature protected from light for 20 min. Complete culture medium (4 ml) was added to the cells and incubated for 5 min to remove excess CFSE. Cells were centrifuged for 5 min at 300*g* at 4 ^°^C, supernatant was removed by decanting, and the cell pellet was resuspended with 1 ml of complete medium. Cells (2 × 10^5^/200 μl) were plated into each well in 96-well plate precoated with anti-CD3 antibody (10 μg/ml) and anti-CD28 (1 μg/ml) (both from BioLegend). Cells were cultured for 72 h and analyzed using a BD LSRFortessa cell analyzer, and data were analyzed using FlowJo software. Proliferation index was calculated as the total number of divisions divided by the number of cells that went into division. Because the proliferation index only takes into account the cells that underwent at least one division, only responding cells are reflected in this value. Division index is the average number of cell divisions that a cell in the original population has undergone. This is an average even for cells that never divided (*i.e.*, includes the undivided peak). Adoptive transfer experiments were carried out for *in vivo* antigen challenge by injecting 0.5 × 10^6^ miR15/16 and OT-I double transgenic CD8+ T cells untouched purified (Miltenyi) into tail veins of C57BL/6J. The following day, ovalbumin SIINFEKL peptide (Sigma) was injected s.c. with 50 μl complete Freund’s adjuvant (Sigma) emulsified with SIINFEKL peptide (20 μg in 50 μl PBS) on day 0 and boosted at day 10 with emulsified incomplete Freund’s adjuvant plus peptide at same concentration. On day 17, mice were sacrificed, and lymph node/spleen cells were analyzed by flow cytometry.

### Statistical analyses

Comparison of two means was carried out using an unpaired Student's *t* test, and three or more groups were analyzed using a one-way ANOVA followed by Tukey’s comparison with GraphPad Prism v.9 (GraphPad, Inc). All comparisons were considered significant at *p* < 0.05. GraphPad Prism was also used to generate standard curves with regression analyses from which values were calculated for sample measurements. For proteomics data, log2 fold change was calculated to determine differences between DOX *versus* PBS groups. The *p* values indicate statistical significance from the replicate samples and were calculated as the significance of each moderated *t* test for each protein being tested. The FDR-adjusted *p* value was calculated to correct for multiple testing.

## Data availability

Project accession number PXD030935 and Web site: https://www.ebi.ac.uk/pride/.

## Supporting information

This article contains supporting information.

## Conflict of interest

The authors declare that they have no conflicts of interest with the contents of this article.
