# Supplementary material for: T-cell activation decreases miRNA-15a/16 levels to promote MEK1–ERK1/2–Elk1 signaling and proliferative capacity
Source: J Biol Chem. 2022 Jan 25;298(3):101634. doi: 10.1016/j.jbc.2022.101634 (PMC8861121; doi:10.1016/j.jbc.2022.101634)
Supplement: Supplemental Figure S1 [file mmc2.pdf]

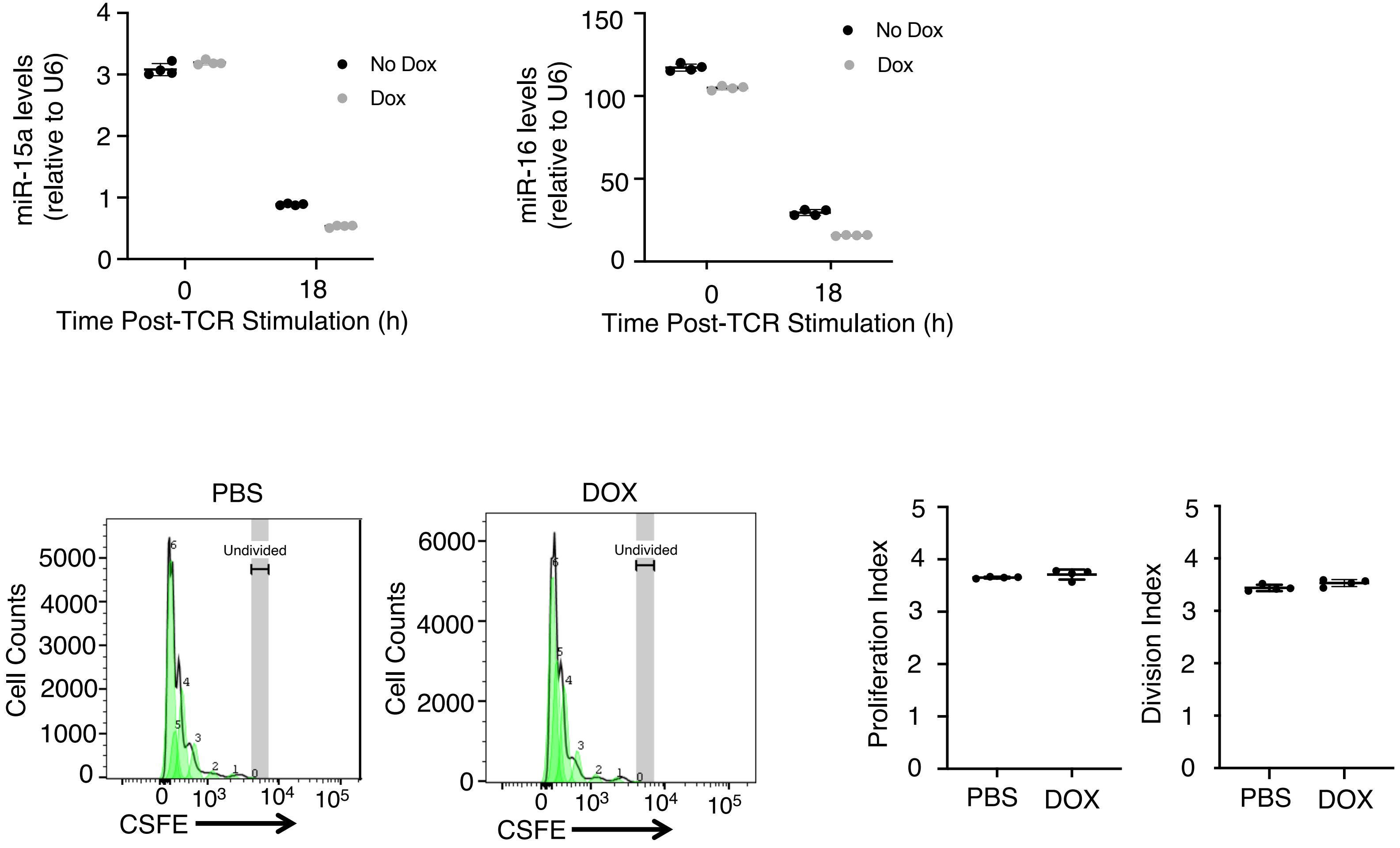

Figure S1. Wild-type C57BL/6 mice without the DOX inducible miR-15a/16 transgene were analyzed for effects of DOX. Levels of miR-15a or miR-16 do not increase with DOX treatment (top panel). CFSE assays analyzed by flow cytometry were used to calculate the Proliferation Index and the Division Index. Data represent mean + S.D.
