# Supplementary material for: T-cell activation decreases miRNA-15a/16 levels to promote MEK1–ERK1/2–Elk1 signaling and proliferative capacity
Source: J Biol Chem. 2022 Jan 25;298(3):101634. doi: 10.1016/j.jbc.2022.101634 (PMC8861121; doi:10.1016/j.jbc.2022.101634)
Supplement: Supplemental Figure S2 [file mmc3.pdf]

			microT-CDS Predicted Interactions for mmu-miR-15a-5p				microT-CDS Predicted Interactions for mmu-miR-15a-3p				microT-CDS Predicted Interactions for mmu-miR-16-1-3p				microT-CDS Predicted Interactions for mmu-miR-16-5p			
	Gene Name	Gene Ensembl id	Gene Name	Gene Ensembl id	Score	Experimentally Supported	Gene Name	Gene Ensembl id	Score	Experimentally Supported	Gene Name	Gene Ensem	Score	Experimentally Supported	Gene Name	Gene Ensembl id	Score	Experimentally Supported
1	Mapk14	ENSMUSG00000053436	Mapk14	ENSMUSG00000053436	0.43	No									Mapk14	ENSMUSG00000053436	0.431	No
2	Vav2	ENSMUSG00000009621	Vav2	ENSMUSG00000009621	0.654	No	Vav2	ENSMUSG00000009621	0.641	No					Vav2	ENSMUSG00000009621	0.652	No
3	Rasgrp1	ENSMUSG00000027347	Rasgrp1	ENSMUSG00000027347	0.409	No	Rasgrp1	ENSMUSG00000027347	0.465	No	Rasgrp1	ENSMUSG0C	0.447	No	Rasgrp1	ENSMUSG00000027347	0.408	Yes
4	Sos2	ENSMUSG00000034801	Sos2	ENSMUSG00000034801	0.851	Yes					Sos2	ENSMUSG0C	0.574	No	Sos2	ENSMUSG00000034801	0.895	Yes
5	Cbl	ENSMUSG00000034342									Cbl	ENSMUSG0C	0.512	No				
6	Cblc	ENSMUSG00000040525					Cblc	ENSMUSG00000040525	0.456	No								
7	Pik3r1	ENSMUSG00000041417	Pik3r1	ENSMUSG00000041417	0.971	Yes					Pik3r1	ENSMUSG0C	0.422	No	Pik3r1	ENSMUSG00000041417	0.969	Yes
8	Pdpk1	ENSMUSG00000024122	Pdpk1	ENSMUSG00000024122	0.505	No	Pdpk1	ENSMUSG00000024122	0.61	No	Pdpk1	ENSMUSG0C	0.471	No	Pdpk1	ENSMUSG00000024122	0.499	No
9	Cd8b1	ENSMUSG00000053044					Cd8b1	ENSMUSG00000053044	0.417	No								
10	Jun	ENSMUSG00000052684					Jun	ENSMUSG00000052684	0.42	No								
11	Pik3r3	ENSMUSG00000028698	Pik3r3	ENSMUSG00000028698	0.436	No	Pik3r3	ENSMUSG00000028698	0.408	No					Pik3r3	ENSMUSG00000028698	0.44	No
12	Gsk3b	ENSMUSG00000022812	Gsk3b	ENSMUSG00000022812	0.795	No	Gsk3b	ENSMUSG00000022812	0.531	No	Gsk3b	ENSMUSG0C	0.84	No	Gsk3b	ENSMUSG00000022812	0.796	No
13	Ppp3cc	ENSMUSG00000022092									Ppp3cc	ENSMUSG0C	0.646	No				
14	Cblb	ENSMUSG00000022637	Cblb	ENSMUSG00000022637	0.442	No					Cblb	ENSMUSG0C	0.468	No	Cblb	ENSMUSG00000022637	0.445	No
15	Ikbkb	ENSMUSG00000031537	Ikbkb	ENSMUSG00000031537	0.747	No									Ikbkb	ENSMUSG00000031537	0.732	No
16	Csf2	ENSMUSG00000018916	Csf2	ENSMUSG00000018916	0.453	No									Csf2	ENSMUSG00000018916	0.453	No
17	Cd28	ENSMUSG00000026012	Cd28	ENSMUSG00000026012	0.921	Yes	Cd28	ENSMUSG00000026012	0.536	No	Cd28	ENSMUSG0C	0.505	No	Cd28	ENSMUSG00000026012	0.893	Yes
18	Malt1	ENSMUSG00000032688	Malt1	ENSMUSG00000032688	0.518	No	Malt1	ENSMUSG00000032688	0.51	No	Malt1	ENSMUSG0C	0.568	No	Malt1	ENSMUSG00000032688	0.514	No
19	Vav3	ENSMUSG00000033721					Vav3	ENSMUSG00000033721	0.416	No								
20	Cd3e	ENSMUSG00000032093	Cd3e	ENSMUSG00000032093	0.548	No									Cd3e	ENSMUSG00000032093	0.551	No
21	Map2k1	ENSMUSG00000004936	Map2k1	ENSMUSG00000004936	0.959	No									Map2k1	ENSMUSG00000004936	0.948	No
22	Ppp3r2	ENSMUSG00000028310					Ppp3r2	ENSMUSG00000028310	0.408	No								
23	Fos	ENSMUSG00000021250					Fos	ENSMUSG00000021250	0.408	No								
24	Nck2	ENSMUSG00000066877									Nck2	ENSMUSG0C	0.526	Yes	Nck2	ENSMUSG00000066877	0.402	No
25	Pik3r2	ENSMUSG00000031834					Pik3r2	ENSMUSG00000031834	0.441	No								
26	Map2k2	ENSMUSG00000035027	Map2k2	ENSMUSG00000035027	0.431	No	Map2k2	ENSMUSG00000035027	0.451	No					Map2k2	ENSMUSG00000035027	0.432	No
27	Tec	ENSMUSG00000029217	Tec	ENSMUSG00000029217	0.608	No					Tec	ENSMUSG0C	0.574	No	Tec	ENSMUSG00000029217	0.613	No
28	Nck1	ENSMUSG00000032475									Nck1	ENSMUSG0C	0.514	No				
29	Grb2	ENSMUSG00000059923	Grb2	ENSMUSG00000059923	0.488	Yes	Grb2	ENSMUSG00000059923	0.416	No					Grb2	ENSMUSG00000059923	0.495	Yes
30	Nfatc3	ENSMUSG00000031902	Nfatc3	ENSMUSG00000031902	0.959	No	Nfatc3	ENSMUSG00000031902	0.432	No					Nfatc3	ENSMUSG00000031902	0.965	Yes
31	Pak1	ENSMUSG00000030774	Pak1	ENSMUSG00000030774	0.481	No									Pak1	ENSMUSG00000030774	0.487	No
32	Lcp2	ENSMUSG00000002699	Lcp2	ENSMUSG00000002699	0.525	Yes	Lcp2	ENSMUSG00000002699	0.453	No					Lcp2	ENSMUSG00000002699	0.511	Yes
33	Rhoa	ENSMUSG00000007815					Rhoa	ENSMUSG00000007815	0.541	No	Rhoa	ENSMUSG0C	0.666	No				
34	Ppp3cb	ENSMUSG00000021816	Ppp3cb	ENSMUSG00000021816	0.575	Yes	Ppp3cb	ENSMUSG00000021816	0.434	No	Ppp3cb	ENSMUSG0C	0.405	No	Ppp3cb	ENSMUSG00000021816	0.59	Yes
35	Akt1	ENSMUSG00000001729					Akt1	ENSMUSG00000001729	0.46	No								
36	Prkcq	ENSMUSG00000026778	Prkcq	ENSMUSG00000026778	0.427	No					Prkcq	ENSMUSG0C	0.546	No	Prkcq	ENSMUSG00000026778	0.425	No
37	Dlg1	ENSMUSG00000022770					Dlg1	ENSMUSG00000022770	0.781	No								
38	Cd8a	ENSMUSG00000053977					Cd8a	ENSMUSG00000053977	0.466	No								
39	Cd4	ENSMUSG00000023274	Cd4	ENSMUSG00000023274	0.447	Yes	Cd4	ENSMUSG00000023274	0.435	No					Cd4	ENSMUSG00000023274	0.426	Yes
40	Kras	ENSMUSG00000030265									Kras	ENSMUSG0C	0.461	No				
41	Mapk3	ENSMUSG00000063065	Mapk3	ENSMUSG00000063065	0.668	No									Mapk3	ENSMUSG00000063065	0.658	No
42	Mapk9	ENSMUSG00000020366	Mapk9	ENSMUSG00000020366	0.702	No	Mapk9	ENSMUSG00000020366	0.482	No					Mapk9	ENSMUSG00000020366	0.706	No
43	4930544G11Rik	ENSMUSG00000036463									4930544G11Rik	ENSMUSG0C	0.577	No				
44	Nfkbie	ENSMUSG00000023947	Nfkbie	ENSMUSG00000023947	0.519	No	Nfkbie	ENSMUSG00000023947	0.558	No					Nfkbie	ENSMUSG00000023947	0.533	No
45	Plcg1	ENSMUSG00000016933	Plcg1	ENSMUSG00000016933	0.498	No	Plcg1	ENSMUSG00000016933	0.556	No					Plcg1	ENSMUSG00000016933	0.487	No
46	Nras	ENSMUSG00000027852	Nras	ENSMUSG00000027852	0.467	No	Nras	ENSMUSG00000027852	0.494	No	Nras	ENSMUSG0C	0.46	No	Nras	ENSMUSG00000027852	0.471	No
47	Map3k14	ENSMUSG00000020941	Map3k14	ENSMUSG00000020941	0.447	No									Map3k14	ENSMUSG00000020941	0.445	No
48	Sos1	ENSMUSG00000024241	Sos1	ENSMUSG00000024241	0.474	No					Sos1	ENSMUSG0C	0.675	No	Sos1	ENSMUSG00000024241	0.478	No
49	Card11	ENSMUSG00000036526	Card11	ENSMUSG00000036526	0.44	No									Card11	ENSMUSG00000036526	0.443	No
50	Map2k7	ENSMUSG00000002948	Map2k7	ENSMUSG00000002948	0.489	No	Map2k7	ENSMUSG00000002948	0.517	No					Map2k7	ENSMUSG00000002948	0.489	No
51	Ifng	ENSMUSG00000055170	Ifng	ENSMUSG00000055170	0.462	Yes									Ifng	ENSMUSG00000055170	0.465	Yes
52	Cd247	ENSMUSG00000005763	Cd247	ENSMUSG00000005763	0.436	No					Cd247	ENSMUSG0C	0.498	No	Cd247	ENSMUSG00000005763	0.441	No
53	Pik3cb	ENSMUSG00000032462					Pik3cb	ENSMUSG00000032462	0.5	No								
54	Hras	ENSMUSG00000025499	Hras	ENSMUSG00000025499	0.414	No	Hras	ENSMUSG00000025499	0.666	No					Hras	ENSMUSG00000025499	0.417	No
55	Chuk	ENSMUSG00000025199									Chuk	ENSMUSG0C	0.429	No				
56	Tnf	ENSMUSG00000024401					Tnf	ENSMUSG00000024401	0.434	No								
57	Nfkb1	ENSMUSG00000028163	Nfkb1	ENSMUSG00000028163	0.578	Yes					Nfkb1	ENSMUSG0C	0.487	No	Nfkb1	ENSMUSG00000028163	0.586	Yes
58	Map3k8	ENSMUSG00000024235	Map3k8	ENSMUSG00000024235	0.434	No									Map3k8	ENSMUSG00000024235	0.424	No
59	Cdk4	ENSMUSG00000006728									Cdk4	ENSMUSG0C	0.464	No				
60	Fyn	ENSMUSG00000019843					Fyn	ENSMUSG00000019843	0.548	No	Fyn	ENSMUSG0C	0.816	No				
61	Ppp3r1	ENSMUSG00000033953									Ppp3r1	ENSMUSG0C	0.434	No				
62	Ppp3ca	ENSMUSG00000028161					Ppp3ca	ENSMUSG00000028161	0.46	No	Ppp3ca	ENSMUSG0C	0.756	No				
63	Ptprc	ENSMUSG00000026395	Ptprc	ENSMUSG00000026395	0.427	No									Ptprc	ENSMUSG00000026395	0.43	No
64	Pdcd1	ENSMUSG00000026285					Pdcd1	ENSMUSG00000026285	0.478	No								
65	Pik3cd	ENSMUSG00000039936					Pik3cd	ENSMUSG00000039936	0.409	No								
66	Rela	ENSMUSG00000024927					Rela	ENSMUSG00000024927	0.509	No								
67	Pak2	ENSMUSG00000022781					Pak2	ENSMUSG00000022781	0.498	No								
68	Map3k7	ENSMUSG00000028284	Map3k7	ENSMUSG00000028284	0.475	No									Map3k7	ENSMUSG00000028284	0.457	No
69	Nfatc2	ENSMUSG00000027544	Nfatc2	ENSMUSG00000027544	0.466	No	Nfatc2	ENSMUSG00000027544	0.486	No								
70	Cdc42	ENSMUSG00000006699	Cdc42	ENSMUSG00000006699	0.487	Yes									Cdc42	ENSMUSG00000006699	0.49	Yes
71	Itk	ENSMUSG00000020395									Itk	ENSMUSG0C	0.529	No				
72	Nfkbib	ENSMUSG00000030595	Nfkbib	ENSMUSG00000030595	0.477	No	Nfkbib	ENSMUSG00000030595	0.416	No					Nfkbib	ENSMUSG00000030595	0.476	No

Figure S2. A complete list of the predicted targets for the four mature miRs generated by the miR-15a/16 transgene. DIANA-miRPath prediction program identified 72 mRNAs potentially targeted by these four miRs with a scores list (1.0 being the strongest).
