# Supplementary material for: T-cell activation decreases miRNA-15a/16 levels to promote MEK1–ERK1/2–Elk1 signaling and proliferative capacity
Source: J Biol Chem. 2022 Jan 25;298(3):101634. doi: 10.1016/j.jbc.2022.101634 (PMC8861121; doi:10.1016/j.jbc.2022.101634)
Supplement: Supplemental Figure S3 [file mmc4.pdf]

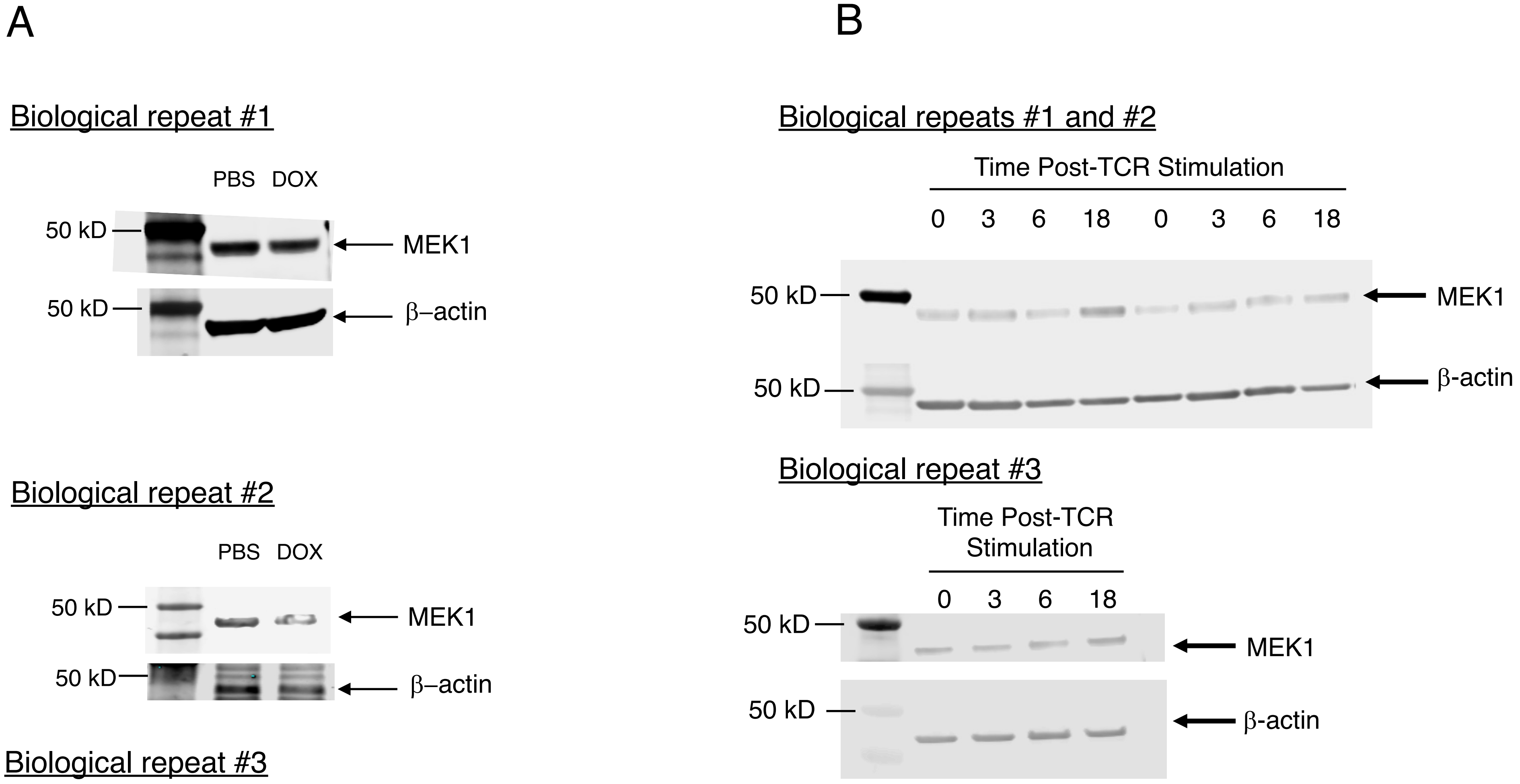

Figure S3. Biological repeat experiments for (A) levels of MEK1 after 18 h TCR-induced activation resulting from DOX-induced expression of miR-15a/16 versus PBS controls, and (B) MEK1 increases at 18 h post-TCR stimulation in WT T cells.
