# Supplementary material for: T-cell activation decreases miRNA-15a/16 levels to promote MEK1–ERK1/2–Elk1 signaling and proliferative capacity
Source: J Biol Chem. 2022 Jan 25;298(3):101634. doi: 10.1016/j.jbc.2022.101634 (PMC8861121; doi:10.1016/j.jbc.2022.101634)
Supplement: Supplemental Figure S4 [file mmc5.pdf]

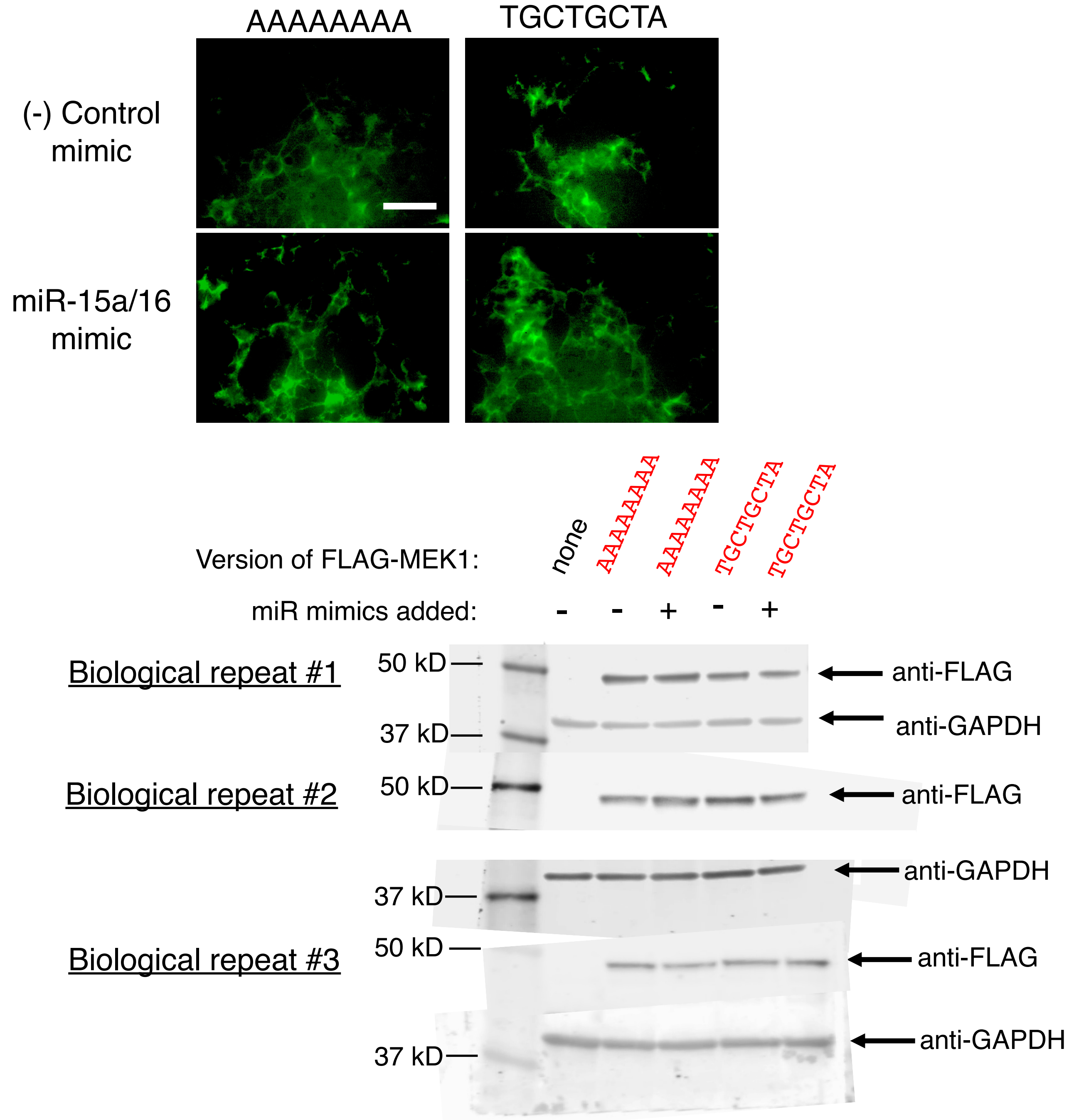

Figure S4. HEK293 cells transfected with miR mimics modified by 5'-fluorescein and 3'-cholesterol. Effective entry of mimics into cells was confirmed by fluorescent microscopy (scalebar = 25 μm), and the western blot replicates used for densitometry in Figure 4 are shown.
