# Supplementary material for: T-cell activation decreases miRNA-15a/16 levels to promote MEK1–ERK1/2–Elk1 signaling and proliferative capacity
Source: J Biol Chem. 2022 Jan 25;298(3):101634. doi: 10.1016/j.jbc.2022.101634 (PMC8861121; doi:10.1016/j.jbc.2022.101634)
Supplement: Supplemental Figure S5 [file mmc6.pdf]

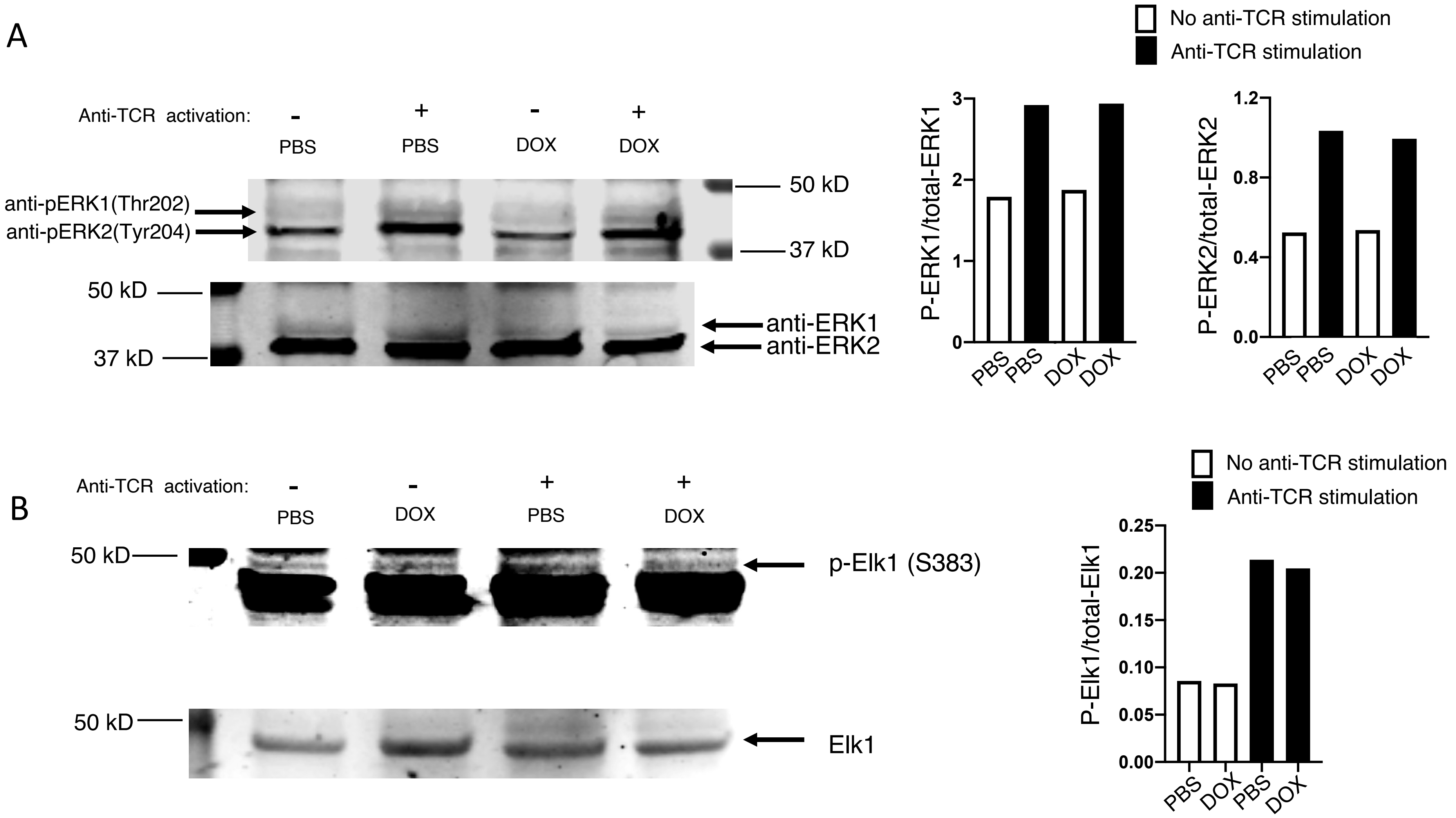

Figure S5. T cells from wild-type C57BL/6 mice without the DOX inducible miR-15a/16 transgene were analyzed for effects of DOX on (A) ERK1/2 and (B) Elk1 in the absence of the DOX inducible miR-15a/16 transgene. WT T cells were activated by anti-CD3/28 for 0 or 18 h in the presence of DOX or PBS as a control. Western blot results show that DOX did not affect levels of phosphorylated and total ERK1/2 (A), or phosphorylated and total Elk1 (B). Data represent mean ± SD.
