# Supplementary material for: T-cell activation decreases miRNA-15a/16 levels to promote MEK1–ERK1/2–Elk1 signaling and proliferative capacity
Source: J Biol Chem. 2022 Jan 25;298(3):101634. doi: 10.1016/j.jbc.2022.101634 (PMC8861121; doi:10.1016/j.jbc.2022.101634)
Supplement: Supplemental Figure S6 [file mmc7.pdf]

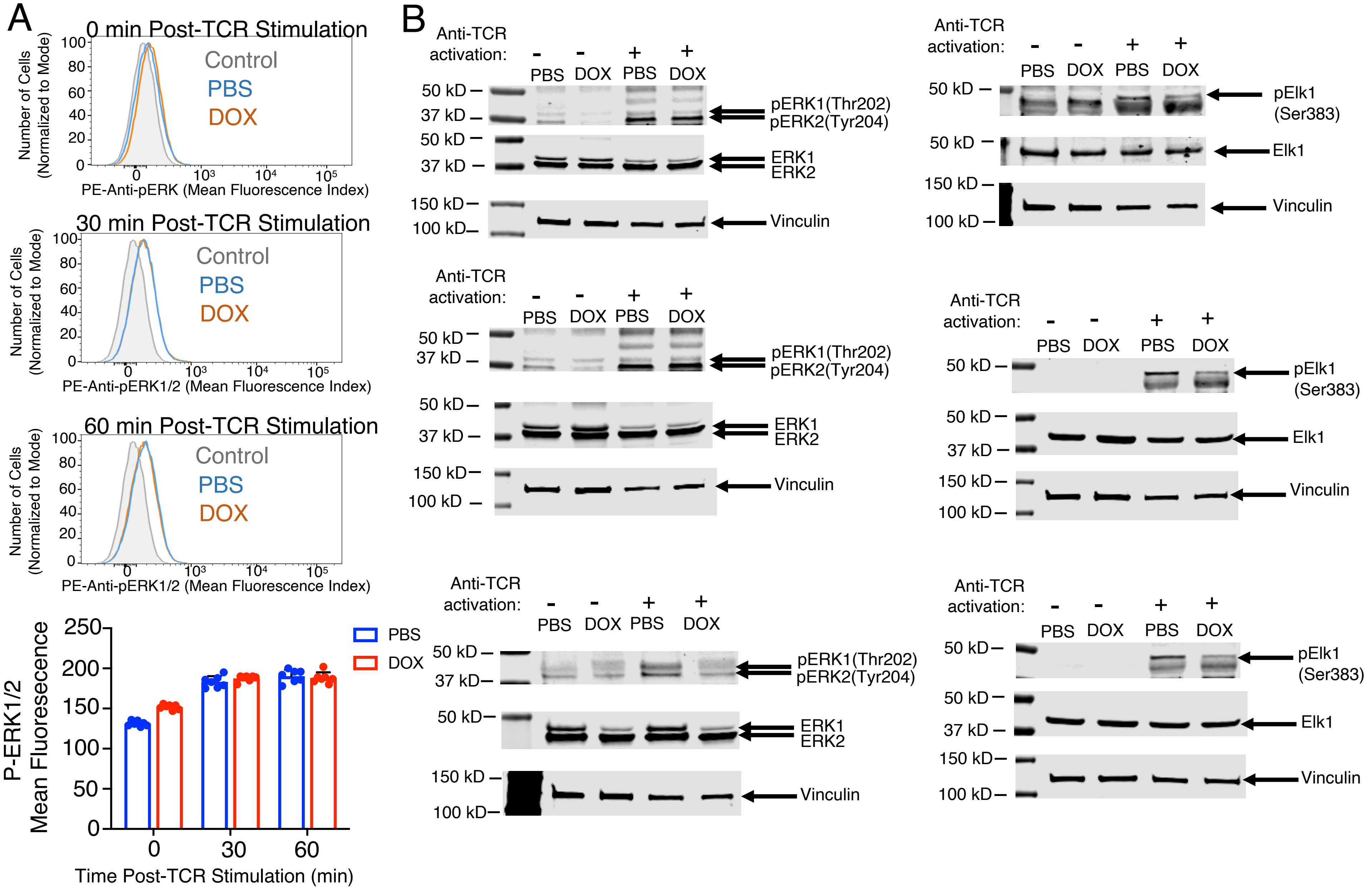

Figure S6. (A) T cells isolated from miR-15a/16 transgenic mice were treated with DOX to induce expression of these miRs, or T cells were treated with PBS as a control. These cells were TCR-stimulated for 0, 30, or 60 min followed by intracellular staining for p-ERK1/2 and analyzed by flow cytometry. Results show similar levels of ERK1/2 phosphorylation between DOX and PBS groups. (B) Three biological repeat experiments for levels of p-ERK1/2 or p-Elk1 after 18 h TCR-induced activation resulting from DOX-induced expression of miR-15a/16 versus PBS controls.
